# Association of Marijuana Use and Cyclic Vomiting Syndrome

**DOI:** 10.3390/ph5070719

**Published:** 2012-06-29

**Authors:** Mithun B. Pattathan, Reza A. Hejazi, Richard W. McCallum

**Affiliations:** Department of Internal Medicine, Paul L. Foster School of Medicine, Texas Tech University Health Sciences Center El Paso, 4800 Alberta Ave, El Paso, TX 79912, USA

**Keywords:** cyclic vomiting syndrome, marijuana, nausea, vomiting, cannabinoid hyperemesis syndrome

## Abstract

Cannabis use has become one of the most commonly abused drugs in the world. It is estimated that each year 2.6 million individuals in the USA become new users and most are younger than 19 years of age. Reports describe marijuana use as high as 40–50% in male Cyclic Vomiting Syndrome patients. It is this interest in cannabis in the World, coupled with recognition of a cyclic vomiting illness associated with its chronic use that beckons a review of the most current articles, as well as a contribution from our own experiences in this area. The similarities we have demonstrated for both cannibinoid hyperemesis syndrome and cyclic vomiting make the case that cannibinoid hyperemesis syndrome is a subset of patients who have the diagnoses of cyclic vomiting syndrome and the role of marijuana should always be considered in the diagnosis of CVS, particularly in males.

## 1. Introduction

Cannabis has become one of the most commonly abused drugs in the World [1]. It is estimated that each year 2.6 million individuals in the USA become new users and most are younger than 19 years of age [2]. In 2008, it was recorded that approximately 2.2 million adolescents aged 12 to 17 in the USA used marijuana for the first time, more than any other illicit drug [3]. These statistics indicate how much this drug is involved with youth society in the United States. It is this interest in cannabis use in the World, coupled with recognition of a cyclic vomiting illness associated with its chronic intake that beckons a review of the most current articles, as well as a contribution from our own experiences. One of our purposes in reviewing the literature is to help organize and present the most current information regarding this particular vomiting syndrome. In this article, will review the current knowledge of how marijuana is linked to the well accepted entity called Cyclic Vomiting Syndrome (CVS) as well as being related to the recently recognized entity of Cannibinoid Hyperemesis Syndrome.

## 2. Methods

A comprehensive PubMed search using such keywords as cyclic vomiting syndrome; nausea; vomiting; trigger factors; marijuana use and cannibinoid hyperemesis syndrome was done and this information was combined with the author’s knowledge and extensive clinical research and publications.

### 2.1. Cannibinoid Hyperemesis Syndrome

Based on our review of the literature, Cannibinoid Hyperemesis Syndrome was first well described and identified in a case series from Adelaide, South Australia by Allen *et al*. where nineteen patients who were chronic cannabis users and among which 80% were males were linked to a cyclical vomiting-like illness [4]. The authors also found that symptoms in nine patients resolved when cannabis use was stopped, as confirmed by urine drug screening. Three patients re-challenged themselves by resuming marijuana and their symptoms re-occurred [4]. Furthermore these patients displayed compulsion patterns of bathing with hot water that remitted their symptoms. The authors concluded that chronic cannabis use must be considered a factor in explaining the cyclical vomiting patterns in their patients. They thought that apart from the possibility of “psychogenic vomiting” no recognized syndrome of vomiting could fit the pattern of their patients.

A recent case series, from Mayo Clinic described 98 patients from 2005 to 2010 with a history of cyclical vomiting with no other explanation for the symptoms except for a history of chronic cannabis use preceding the symptoms [5]. Eighty-four patients reported abdominal pain and fifty-two patients reported improvement of symptoms after bathing with hot water in a bath or shower. Seven patients who were followed up stopped marijuana use and six out those seven noted complete resolution of their vomiting episodes [5].

Another case report described a 22 year old male with a 6 year history of marijuana use who had recurrent episodes of nausea, refractory vomiting, and colicky epigastric pain [6]. This patient had been seen twice previously in emergency departments for nausea and vomiting in the past two months [6] and noted that taking hot baths aided in relief of his symptoms. He was treated with intravenous fluids, metoclopramide, pantoprazole, and morphine for the abdominal pain. Laboratory values and imaging did not reveal a specific etiology. Diagnosis was made that his nausea and vomiting might be attributed to his use of marijuana. After cessation of marijuana use, the patient reported that his symptoms resolved completely and he did not have any more episodes of nausea and vomiting [6].

### 2.2. Cyclic Vomiting Syndrome

Over the years there has been recognition of a pediatric Cyclical Vomiting Syndrome (CVS). It was first described in 1882 by Samuel Gee, who reported this condition in a series of nine children [7]. Since that first publication, numerous other reports in the pediatric literature have illustrated how much more common this condition is becoming. In the pediatric setting migraine headaches are regarded as an important component of the attacks. In 1988, Abell *et al*. [8] described a series of eight adult patients with CVS. Since these cases the number of reports of CVS in adults has reached a point where over the last 10 years it is more dominant than its prevalence in the pediatric population [9], which had always been considered as the patients at risk for CVS.

In adults, severe abdominal pain accompanies the nausea and vomiting cycles and migraine headaches are only thought to be contributing in approximately 30% of the group, but they were a major potential trigger in pediatric patients and marijuana was never a consideration in the pediatric age group. This dramatic increase in the recognition and diagnosis of CVS in adults has resulted in major changes in the way nausea and vomiting is viewed in the adult population. About 3–14% of patients referred for unexplained nausea and vomiting to University Medical Centers in the US have CVS as the etiology [10].

Cyclic vomiting is a syndrome characterized with recurrent episodes of incapacitating nausea and vomiting interspersed with relatively symptom-free intervals lasting anywhere from a few days to several months. According to Rome III the following criteria must be included to diagnose the illness (Table 1): (1) stereotypical episodes of vomiting regarding onset (acute) and duration (less than 1 week); (2) three or more discrete episodes in the previous year; (3) absence or infrequency of nausea and vomiting between episodes. These criteria must be fulfilled for the last three months with symptom onset at least six months before diagnosis can be achieved [10].

**Table 1:** Rome III criteria [10].

**A.**	Stereotypical episodes of vomiting regarding onset (acute) and duration (less than 1 week)
**B.**	Three or more discrete episodes in the previous year
**C.**	Absence or infrequency of nausea and vomiting between episodes
**D.**	These criteria must be fulfilled for the last 3 months with symptom onset at least 6 months before diagnosis can be achieved

Reviewing the literature of adult Cyclic Vomiting Syndrome it was revealed that approximately 42–53% of patients were marijuana users [10]. In one report only about 5 percent of chronic users viewed marijuana as the cause of their vomiting cycles [10]. There is a high predominance of males among the marijuana users. In addition it is common to have a history of seeking relief with hot showers or baths [4,5,6,11]. Marijuana smoking began typically in their teenage years for recreational reasons and vomiting did not start until at least 5 and often more than 10 years later. The age of onset of symptoms usually ranges from 20 to 40 years of age. The CVS patients were continuing to smoke almost daily up to the time of diagnosis of CVS. On the other hand, marijuana can also be used by CVS patients intermittently for alleviating nausea/vomiting symptoms. This is a different group than those chronically smoking marijuana for years.

The significance of marijuana as a causative or triggering agent in CVS is still not established [11]. Withdrawing marijuana in our experience is recommended over time, but only after the typical therapy has been initiated. Tricyclic antidepressant (TCA) use, specifically amitriptyline in doses of up to 100 to 200 mg at night, as well as anti-anxiety medications (*i.e*., lorazepam) are the standard therapy for CVS. This provides chemical stability and prevents the possibility of repeated episodes of vomiting due to marijuana withdrawal. *Namin et al.*, in a study of cyclic vomiting involving 31 adult patients, found that 13 patients used marijuana in their series; seven had symptom relief, while two had resolution of CVS after stopping use [12]. Treatment experience in 24 of those patients receiving amitriptyline up to 1 mg/kg/day for at least three months indicated that 93% had decreased symptoms and 26% achieved full resolution [12]. Cessation of marijuana is best attempted while on TCA and/or anti-anxiety medications when symptom control has been achieved. In addition, cannabis has been demonstrated to delay gastric emptying acutely, which could contribute to initiation of a relapse [13,14,15]. Therefore we conclude from the analysis of the literature that great similarities exist between the unexplained cycles of vomiting in marijuana patients and the well established syndrome of CVS (Table 2). Specifically:

(A) Marijuana use precedes most cyclic vomiting attacks by years. Typically beginning as recreational use in teenage years and then years later the vomiting illness evolves;
(B) Association of hot baths/showers for symptomatic relief;
(C) Abrupt attacks with accompanying abdominal pain and emergency room visits and hospitalization;
(D) Diagnosis considered after extensive work up is negative for other explanations.

**Table 2:** Cannabinoid hyperemesis syndrome: Proposed criteria [16].

**Essential**	History of long term cannabis use
**Major Features**	1. Cyclic episodes of nausea and vomiting
	2. Reduction and/or resolution of symptoms after v cannabis cessation
	3. Relief of symptoms with hot showers or baths
	4. Abdominal pain (epigastric or periumbilical)
	5. History of frequent-even daily use of marijuana
**Supportive Features**	1. Males dominate
	2. Patients usually less than 40 years of age
	3. Negative laboratory, radiographic and endoscopic test results.
	4. Weight loss not a prominent feature

### 2.3. Cyclic Vomiting and Migraines

The association between Cyclic Vomiting Syndrome and migraines has been described in the literature. This association has been considered since 1904, as mentioned by Rachford [17]. In a retrospective study, 35 children (17 males, 18 females) previously diagnosed with cyclic vomiting syndrome were enrolled and their symptoms observed. Of these 35 children, 20 developed symptoms of migraines [18]. Conclusions of this study were that there may be an increased risk of migraine development in CVS patients who had symptoms that began at a younger age of onset and had headaches during their CVS attacks. In about 24–70% of adult patients affected by cyclic vomiting syndrome a history or family history of migraines was reported [10]. In pediatric patients with a history or family history of migraines it has been reported as around 39–82%. In another larger study Li *et al*. reported 214 patients with a family history and/or history of migraine attacks and found about 82% of the cases were thought to be migraine-associated cyclic vomiting [19]. In 1993, the possibility was raised that there may be a genetic association between migraines and CVS [16]. A study conducted at the University of Kansas, demonstrated that mitochondrial polymorphisms occurred mostly in the pediatric-onset CVS patients as opposed to the adult-onset CVS population [20]. There was an increased prevalence in both groups with occurrence of migraines as opposed to the control group. With the increased association between migraines and CVS, the concept of using medications that treat the former to help alleviate the latter has been considered. In one case report, a 47 year old male diagnosed with CVS had his symptoms remit successfully with intranasal sumatriptan [21]. In another case report, a 14 year old female with 18q syndrome (a rare genetic disorder involving the deletion of the long arm of chromosome 18) and previously diagnosed with pediatric-onset CVS was treated successfully with sumatriptan during her attacks [22].

### 2.4. Theories

How can we explain the connection between cannabis use and cyclic vomiting? Delta-9-tetrahydrocannabinol (THC), the chief main active cannabinoid (CB) binds to CB-1 and CB-2 receptors in human tissues. CB-1 receptors are located in the central nervous system and enteric nervous system and CB-2 receptors are located mostly in immune tissue. It is primarily the CB-1 receptor that is thought to be involved with anti-emetic effects that cannabis use has often been linked to in regards to treating cancer patients [23]. It is considered that sustained stimulation of this receptor based on dose-dependent THC can slow gastrointestinal motility and could be a culprit in inducing the symptoms as mentioned in one article [13]. It has been shown that an intravenous injection of crude marijuana extract has the capacity to induce vomiting [24]. Marijuana is thought to work on the limbic system of the brain, particularly at the hippocampal-hypothalamic-pituitary level [4]. It is thought that marijuana may affect satiety, thirst, digestive, and thermoregulatory systems of the hypothalamus. This disruption might be reduced or settled with hot bathing or showering, as indicated by many patients [11,25,26].

What is the exact time-dependent duration of cannabis use that leads to these symptoms has not been properly chronicled in any current literature. Also the number of patients affected is probably only a small subset of the population of those engaged in chronic marijuana use. This raises several questions about the possibility that other factors such as genetic receptor makeup, psychological status, and total dose of marijuana intake may contribute to the patients’ condition. Crude marijuana is made up of over 60 different compounds plus there is the possibility of contamination with chemicals and toxins from handling of the marijuana that may accumulate in the brain and also pose as factors that could contribute to the patients’ condition. It is known that THC is lipophilic and binds to cerebral fat and has a long half life. This could be a factor contributing to the subset of cannabis users who develop vomiting that cannabinoid receptor function maybe related to such factors as body size and habitus may. It is unknown if chronic marijuana use has a deleterious effect on the endogenous cannabinoid system or if other constituents of the inhaled smoke from marijuana cigarettes could accumulate in toxic quantities [27].

### 2.5. Management of Cyclic Vomiting with Concomitant Marijuana Use

Many anti-emetic medications including ondansetron, prochlorperazine, metoclopramide, and promethazine, have some effect in relieving nausea and vomiting at initial presentation [28]. Intravenous lorazepam has shown to be the “key” in the emergency room by sedating the patient. Published case reports have demonstrated that intravenous hydration can improve the patients’ condition and that a physician should inspect for signs of volume depletion on examination [12,29]. Tricyclic antidepressants (TCA) are shown to be effective in several studies for long-term prophylaxis [30]. Initially a low-dose of TCA, the most popular being amitriptyline, is titrated to to a higher dose level achieve the desired effect [7]. This regimen will sustain symptom relief and permit cessation of marijuana use. The hope is that most patients that have CVS and also engage in marijuana use will have symptoms remit and TCA dosing can be tapered. Marijuana use can also adversely affect the response to standard tricyclic antidepressant therapy for CVS. In a recent study, chronic marijuana use was reported in 53% of the adult CVS patients who were not responded to TCA therapy *vs*. 22% in the responder group [31]. Marijuana use by these patients was perceived as therapeutic for improving symptoms but was in retrospect it was inducing CVS episodes.

However there are questions about the marijuana relationship that remains unanswered: (1) why do patients smoking marijuana every day only have dramatic attacks of nausea and vomiting and abdominal pain every few weeks? Why not a more daily occurrence if they smoke everyday? (2) Once sensitized the CB receptors of marijuana could take months to really change from years of smoking so symptoms could take some time to really abate once marijuana use is stopped; (3) Could stopping marijuana use abruptly lead to a withdrawal effect with vomiting and thus mimic a CVS attack. For now, our recommended approach is to strongly advise for cessation of marijuana use in adult CVS with replacement by alternative “relaxation” approaches including relaxation techniques while continuing overall management with TCA, lorazepam, anti-emetic, and non-narcotic pain medications. Over time a slow tapering of the tricyclics can be attempted.

## 3. Conclusions

Cyclic Vomiting Syndrome is a disorder characterized by recurrent episodes of severe nausea and vomiting and abdominal pain separated by symptom-free periods. Its relationship with cannabis use is becoming increasingly recognized in the world. Patients affected by cyclic vomiting-illness must be questioned about their social and recreational behavior as well. The similarities we have demonstrated for both cannibinoid hyperemesis syndrome and cyclic vomiting make the case that cannibinoid hyperemesis syndrome is a subset of patients who have the diagnoses of cyclic vomiting syndrome and the role of marijuana should always be considered in the diagnosis of CVS-particularly in males. Cessation of marijuana is encouraged to reduce and hopefully halt the cycles. All physicians particularly in the Emergency Department in particular should be alerted to this clinical setting.
